# Experimental Investigation on the Performance of Full Tailings Cemented Backfill Material in a Lead–Zinc Mine Based on Mechanical Testing

**DOI:** 10.3390/ma19020351

**Published:** 2026-01-15

**Authors:** Ning Yang, Renze Ou, Ruosong Bu, Daoyuan Sun, Fang Yan, Hongwei Wang, Qi Liu, Mingdong Tang, Xiaohui Li

**Affiliations:** 1 School of Resources and Safety Engineering, Central South University, Changsha 410083, China; 225502039@csu.edu.cn (N.Y.); 255502047@csu.edu.cn (R.O.); 255512021@csu.edu.cn (R.B.); sundaoyuan@csu.edu.cn (D.S.); whuwhw@whu.edu.cn (H.W.); 2 Changsha Institute of Mine Research Co., Ltd., China Minmetals Corporation, Changsha 410083, China; liuqicsy@163.com (Q.L.); tangmd6@minmetals.com (M.T.); lixiaohui@cumt.edu.cn (X.L.)

**Keywords:** mining engineering, cemented backfill composite, failure mode, uniaxial compression test

## Abstract

With the increasing requirements for “Green Mine” construction, Cemented Tailings Backfill (CTB) has emerged as the preferred strategy for solid waste management and ground pressure control in underground metal mines. However, full tailings, characterized by wide particle size distribution and high fine-grained content, exhibit complex physicochemical properties that lead to significant non-linear behavior in slurry rheology and strength evolution, posing challenges for accurate prediction using traditional empirical formulas. Addressing the issues of significant strength fluctuations and difficulties in mix proportion optimization in a specific lead–zinc mine, this study systematically conducted physicochemical characterizations, slurry sedimentation and transport performance evaluations, and mechanical strength tests. Through multi-factor coupling experiments, the synergistic effects of cement type, cement-to-tailings (c/t) ratio, slurry concentration, and curing age on backfill performance were elucidated. Quantitative results indicate that solids mass concentration is the critical factor determining transportability. Concentrations exceeding 68% effectively mitigate segregation and stratification during the filling process while maintaining optimal fluidity. Regarding mechanical properties, the c/t ratio and concentration show a significant positive correlation with Uniaxial Compressive Strength (UCS). For instance, with a 74% concentration and 1:4 c/t ratio, the 3-day strength increased by 1.4 times compared to the 68% concentration, with this increment expanding to 2.0 times by 28 days. Furthermore, a comparative analysis of four cement types revealed that 42.5# cement offers superior techno-economic indicators in terms of reducing binder consumption and enhancing early-age strength. This research not only establishes an optimized mix proportion scheme tailored to the operational requirements of the lead–zinc mine but also provides a quantitative scientific basis and theoretical framework for the material design and safe production of CTB systems incorporating high fine-grained full tailings.

## 1. Introduction

### 1.1. Research Background

With the increasing depth of global mineral resource extraction and the implementation of more stringent environmental policies, the construction of “green mines” has become a core requirement for the sustainable development of the mining industry. Among various mining methods, backfilling has become the preferred solution for underground metal mines due to its effectiveness in controlling ground pressure, improving resource recovery rates, and mitigating safety hazards associated with surface tailings dams. As the core technology of this method, the performance of Cemented Tailings Backfill (CTB) directly dictates the operational safety and economic efficiency of the mine.

However, traditional cemented backfilling often requires the classification of tailings to remove the fine-grained fractions. This practice not only leads to a low utilization rate of tailings and difficulties in disposing of the remaining fine particles but also increases the processing burden of the mineral dressing plant. Cemented Tailings Backfill (CTB), as a more environmentally friendly and efficient technical alternative, enables the total recycling of all mill tailings, thereby minimizing surface discharge. Nevertheless, full tailings are characterized by a broad particle size distribution, high fine-particle content, and complex physicochemical properties. Consequently, the rheological behavior, sedimentation stability of the slurry, and the mechanical strength of the hardened body are influenced by multiple factors, exhibiting distinct nonlinear characteristics.

### 1.2. Research Status

Significant research has been conducted on the performance evolution and optimization of cemented backfill materials. Regarding basic mechanical properties, Li and Jia [1] investigated the mechanical behavior of backfill under multiaxial compression, providing a baseline for stability assessment in complex ground pressure environments. Liu and Fall [2] further tested various properties of cemented backfill under multiaxial compressive loading, confirming the significant impact of the stress environment on its strength. In terms of material composition optimization, Jiang et al. [3] examined the effect of using recycled concrete powder as a sand replacement on mortar properties, while Garcia-Troncoso et al. [4] analyzed the mechanical suitability of mining tailings as fine aggregates in concrete through comparative experiments.

As research progressed, the influence of multi-factor coupling on the performance of Cemented Tailings Backfill (CTB) became a focal point. Zhang et al. [5] systematically compared the effects of different binders on the workability, mechanical strength, and microstructure of fine tailings backfill. Hou et al. [6] achieved a synergistic improvement in both workability and strength of CTB by incorporating mineral admixtures and fibers. For specific operating conditions, Zhang et al. [7] analyzed the stability and optimized the strength of backfill in high-temperature mines. Furthermore, Samiratou Yaya et al. [8] explored the influence of 3D-printed skeleton shapes on the strength behavior and stress evolution of backfill materials.

In the field of microscopic damage and evolution mechanisms, Gao et al. [9] utilized ultrasonic time-frequency response characteristics to reveal the damage evolution patterns of backfill concrete under staged loading. Bai et al. [10] systematically elaborated on the mechanical characteristics and microstructural properties of CTB based on uniaxial compression tests. Wu et al. [11] further established a quantitative relationship between crack initiation stress and uniaxial compressive strength (UCS) for cemented waste rock-tailings backfill. Additionally, focusing on the layered structures commonly found in actual stopes, Chen et al. [12] conducted an in-depth study on the strength deterioration mechanism of horizontally stratified CTB under uniaxial compression, providing a theoretical basis for quality control in underground backfilling operations.

In the field of international research, extensive explorations have been conducted on the diversification and performance enhancement of backfill materials. Regarding raw material innovation, Al-Bakri et al. [13] experimentally investigated the feasibility of using cement kiln dust (CKD) as a supplementary cementitious material for copper tailings cemented backfill. Yuan, Q et al. [14] introduced thermally and mechanically treated natural pozzolans to improve the mechanical properties of copper tailings backfill. Concerning the influence of aggregate characteristics, Guner et al. [15] explored the effects of partially replacing copper-rich tailings with sand on the rheology, strength, and microstructure of the backfill. Yin et al. [16] studied the synergistic effects of aggregate size distribution and carbon nanotubes on the mechanical performance of gangue cemented backfill under true triaxial compression.

To further enhance early-age strength and engineering performance, several innovative curing and additive technologies have been proposed. Hefni [17] presented an innovative method using microwave-assisted curing to enhance the early strength of cemented backfill. Cavusoglu and Fall [18] systematically evaluated the impact of polycarboxylate-based superplasticizers on the engineering properties of cemented backfill. Furthermore, addressing mechanical responses under specific service environments, Safari et al. [19] investigated the microstructural behavior of cemented backfill under cyclic loading. Behera et al. [20] compared the tensile strength differences of lead–zinc tailings backfill between laboratory and in situ scenarios, providing a practical reference for underground design. Chiloane et al. [21] combined experiments with numerical simulations to reveal the complex patterns of strength development in layered backfill.

In recent years, nonlinear response prediction and intelligent methodologies have emerged as new research trends. Jafari et al. [22] developed a method to predict the nonlinear mechanical response of cemented backfill to mining-induced closure strains. Mishra et al. [23] explored the reinforcement mechanisms of polypropylene fibers on the mechanical and microstructural properties of coal ash-based backfill. Simultaneously, with the advancement of data science, Shaaban et al. [24] attempted to utilize machine learning algorithms to predict the compressive strength of high-strength concrete, offering a new methodology for backfill strength prediction. Despite the progress made in material modification and performance evaluation, systematic and in-depth experimental research remains essential to address the compatibility between specific full tailings compositions (such as the lead–zinc mine in this study) and local cementitious materials.

### 1.3. Research Objective

To reduce the economic costs of cemented tailings backfilling for goafs, mining enterprises often adopt layered backfilling with cemented backfills of varying cement-to-tailings (c/t) ratios. The synergistic deformation mechanism and mechanical properties between backfills with different c/t ratios directly determine the goaf backfilling effect. Based on slump tests and L-shaped pipeline transportation tests, this study selected unclassified tailings slurries with a mass concentration range of 68–74% and systematically conducted strength tests on backfills under different c/t ratios (1:4 to 1:15) and cement grades (32.5# and 42.5#). Small-scale pipeline transportation simulation tests were carried out to simulate the pipeline conveyance process of unclassified tailings slurries with different concentrations, and the transportation performance of the slurries was analyzed and calculated based on the test data.

The core purpose of this study is to provide backfilling process parameters for the safe production of mines and offer a reliable theoretical basis and technical reference for the design of backfill mining. Specifically, it aims to accurately address the engineering pain points of a lead–zinc mine’s unclassified tailings, such as significant fluctuations in backfill strength and difficulties in mix proportion optimization, which are caused by wide particle size distribution and high fine-grained content. Meanwhile, targeting the practical issues of high cement transportation costs and unclear applicability of different grade cements in the mine, this study verifies the feasibility of Cemented Tailings Backfill (CTB) technology in reducing backfilling costs and ensuring goaf stability through systematic comparison of the techno-economic efficiency of cements from different brands and strength grades. The research results not only directly guide the efficient, safe, and economical backfilling practices of the lead–zinc mine, providing key technical support for its “Green Mine” construction, but also offer a scientific mix proportion design scheme, clear process parameter range, and solid theoretical and technical reference for the resource utilization of unclassified tailings in metal mines with similar physical properties.

## 2. Raw Materials and Experimental Methods

### 2.1. Backfill Test Materials

The cement types selected for the tests include Kunlunshan PO42.5 (produced by Xinjiang Tianshan Cement Co., Ltd., Urumqi, China), Jinyuan PC32.5R (manufactured by Jinyuan Cement Co., Ltd., Hangzhou, China), Baishushan PO42.5 (produced by Hunan Baishushan Cement Group Co., Ltd., Changsha, China), and Baishushan PC32.5R (manufactured by Hunan Baishushan Cement Group Co., Ltd.). According to statistics from the mine where backfilling is implemented, the cost of cement accounts for more than 65% of the total backfilling cost, making it a key factor in backfilling cost control. Considering the long transportation distance of cement for the A Lead–Zinc Mine (Xitieshan Branch of Western Mining Co., Ltd., Xitieshan, China), which results in high transportation costs and poor stability of cement quality in the region, coupled with the minimal cost difference between 32.5# and 42.5# cement—even in some cases, the delivered price of 32.5# cement is higher than that of 42.5# cement—42.5# cement offers distinct advantages such as lower dosage, higher early strength, and a wider range of backfill strength coverage under the same strength requirements. Therefore, to select cement with high cost-effectiveness for the tests, two types of 32.5# cement and two types of 42.5# cement were determined in collaboration with the mine. The backfill aggregate used is unclassified tailings from the concentrator of the A Lead–Zinc Mine, which were sampled and shipped to Changsha by the mine.

### 2.2. Basic Physical Parameters and Chemical Composition of Unclassified Tailings

#### 2.2.1. Physical Parameters of Unclassified Tailings

The determination of physical parameters of unclassified tailings primarily includes specific gravity, loose bulk density, compacted bulk density, and the porosity calculated from specific gravity and bulk density.

Specific gravity of tailings: Measured using the pycnometer method. Specifically, a standard pycnometer is first weighed empty, then filled with water to the standard calibration line, and the total weight of the pycnometer and water is recorded. Next, a portion of the water is removed, and a predetermined mass of dry tailings is added to the pycnometer containing the remaining water. After shaking to ensure homogeneity, distilled water is added to reach the standard calibration line. The mixture is allowed to soak for 24 h. Due to minor evaporation, the liquid level may drop; thus, distilled water is replenished to the calibration line. The total weight is then measured, and the specific gravity of the tailings at room temperature is calculated accordingly.

Bulk Density of Tailings: The loose bulk density is determined using the constant-volume weighing method. A standard funnel is filled with tailings, and a standard one-liter container is placed underneath. The material is allowed to fall freely into the container without vibration. Once the container is full, the surface is leveled, and the weight is recorded. The measurement is repeated several times to calculate the average loose bulk density.

The compacted bulk density is also measured via the constant-volume weighing method. Similarly, tailings are placed in a standard funnel above a standard one-liter container. During filling, the container is continuously vibrated to achieve compaction. After the container is filled and leveled, the weight is recorded. Multiple trials are conducted to compute the average compacted bulk density. The test results of tailings specific gravity, bulk density, and the calculated porosity are presented in Table 1.

#### 2.2.2. Chemical Composition

Various chemical components and their contents in backfill materials exert certain influences on the physical properties of cemented backfill. The main chemical elements in tailings that significantly affect backfill strength include Ca, Mg, Al, Si, and S. Among these, the content of sulfides has the most prominent impact on the performance of cemented backfill. The sulfur content in the tailings is 1.33% (<6%), which exerts little adverse effect on the backfill. A relatively high Si content is conducive to enhancing the backfill strength.

#### 2.2.3. Particle Size Distribution of Unclassified Tailings

**Figure 1:**
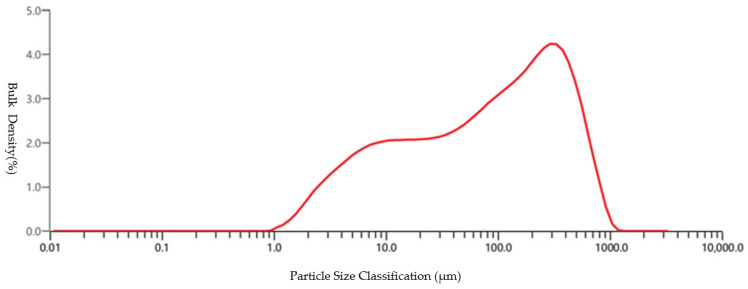
Particle Size Distribution Curve of Tailings.

The particle size composition of tailings exerts a significant influence on mine backfilling, with key parameters including median diameter and coefficient of particle uniformity. In this study, a MASTERSIZER laser diffraction particle size analyzer [Malvern Panalytical Ltd., Malvern, UK] was employed for particle size analysis, and the test result is presented in Figure 1.

### 2.3. Test Block Mix Ratio and Strength Optimization Test

#### 2.3.1. Specimen Preparation

Standard 7.07 cm × 7.07 cm × 7.07 cm triple molds [Cangzhou Luyi Testing Instrument Co., Ltd., Cangzhou, China] were used, with their inner walls pre-coated with lubricating or machine oil for easy demolding. Backfill materials were measured by mix ratio: cement and tailings weighed via electronic balance, and water measured with a graduated cylinder. Weighed materials were poured into a mixer, mixed with specified water (per target mass concentration) first manually, then mechanically to form uniform slurry. The slurry was poured into molds, with multiple replenishments after natural settlement to ensure full filling with slight excess. After standing, the slurry’s surface was screeded once initially set. Demolding was performed when blocks gained self-support, and demolded blocks were immediately transferred to a curing pool (95% humidity, 20 °C) for maintenance.

#### 2.3.2. Uniaxial Compressive Strength Test: Methods and Equipment

**Figure 2:**
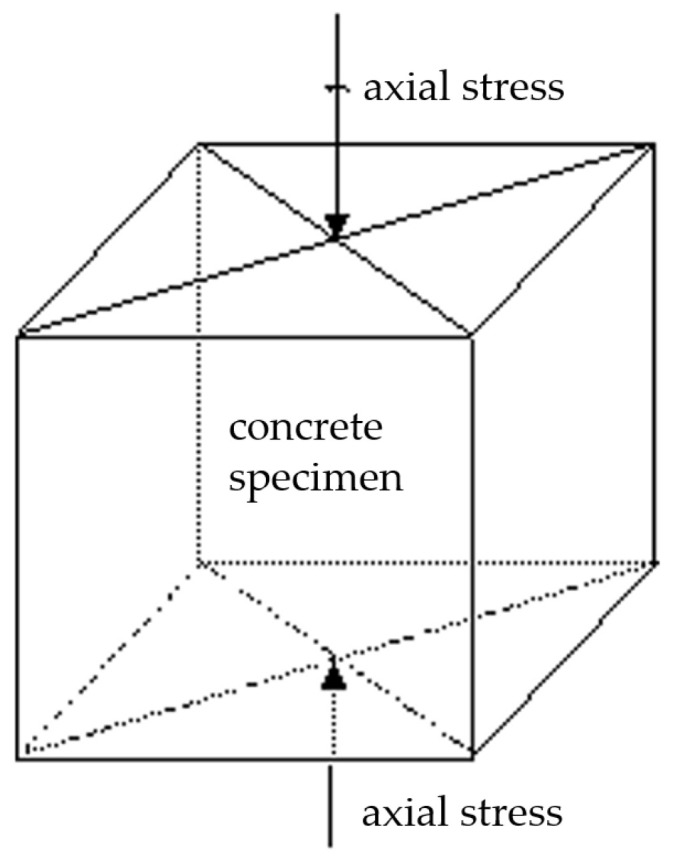
Schematic Diagram: force diagram of uniaxial compressive strength test.

The stress state of the tested specimens during the experiment is shown in Figure 2. A NYL-300D pressure testing machine [Jinan Zhongluchang Testing Machine Manufacturing Co., Ltd., Jinan, China] was used to determine their uniaxial compressive strength. For each curing age, three test blocks were selected for the experiment, and their uniaxial compressive strengths were measured. The average value was taken as the uniaxial compressive strength of the backfill at that age.

#### 2.3.3. Mix Proportion Test Design of Backfill Specimens

The test design for cement-bonded unclassified tailings slurry specimens is presented in Table 2. Based on the results of slump tests and L-type pipeline transportation simulation tests, four types of cement, four concentrations (74%, 72%, 70%, 68%), five cement–sand ratios (1:4, 1:6, 1:10, 1:12, 1:15), and five curing ages (3 days, 7 days, 14 days, 28 days, 60 days) were selected for a comprehensive combinatorial test. Consequently, a total of 400 groups of specimens need to be prepared. Since each test requires at least three specimens to be tested, the total number of specimens required is 1200. The backfill specimens with different mix proportions were cured at the specified curing ages (3 days, 7 days, 14 days, 28 days, 60 days). After reaching the respective curing ages, the specimens were removed from the curing tank, followed by the determination of their uniaxial compressive strength values.

### 2.4. Determination of Slump of Backfill Slurry

The experimental materials used in this study comprised full tailings obtained from a processing plant of a lead–zinc mine, three brands of Portland cement (Jinyuan 32.5#, Kunlunshan 42.5#, Baishushan 32.5#, and Baishushan 42.5#), and domestic water. The experimental design was centered on evaluating the pipeline transportability of full tailings cemented backfill slurry through a multi-factor variable approach, encompassing four binder combinations, one control group (pure tailings), and five cement-to-sand ratios (1:4, 1:6, 1:10, 1:12, and 1:15). Regarding mass concentration control, the tests commenced at an initial concentration of 84% and decreased in 2% increments until the slurry reached an ultimate spread-out state.

The workability, cohesiveness, and water retention of the slurry were tested in strict accordance with the Standard for Test Methods of Performance on Ordinary Fresh Concrete (GB/T 50080-2016) [25]. A standard slump cone with an upper diameter of 100 mm, a lower diameter of 200 mm, and a height of 300 mm was employed. The prepared slurry was poured into the cone in three uniform layers, with each layer being tamped 25 times using a tamping rod to ensure compaction. The entire operation, from filling to lifting the cone, was completed within 150 s, with the lifting process controlled between 5 and 10 s. The slump value, defined as the height difference between the top of the cone and the highest point of the collapsed slurry, was measured as a quantitative indicator of slurry fluidity.

Simultaneous with the slump data recording, the cohesiveness and water retention of the slurry were comprehensively evaluated via visual inspection. Particular focus was placed on observing whether the slurry maintained its structural integrity after collapsing and whether phenomena such as aggregate stratification, significant edge bleeding, or segregation occurred. Ultimately, a dataset of slump evolution under different cement-to-sand ratios, cement types, and concentrations was obtained. Combined with the apparent morphology of the slurry, the critical concentration range satisfying the requirements for pipeline transportation in the specific mine was identified. These precise physical parameters provided theoretical support for analyzing the weighting of various factors influencing backfill performance.

## 3. Experimental Results and Analysis

### 3.1. Compressive Strength of Filling Body Test Block

#### 3.1.1. Jinyuan 32.5# Cement

**Figure 3:**
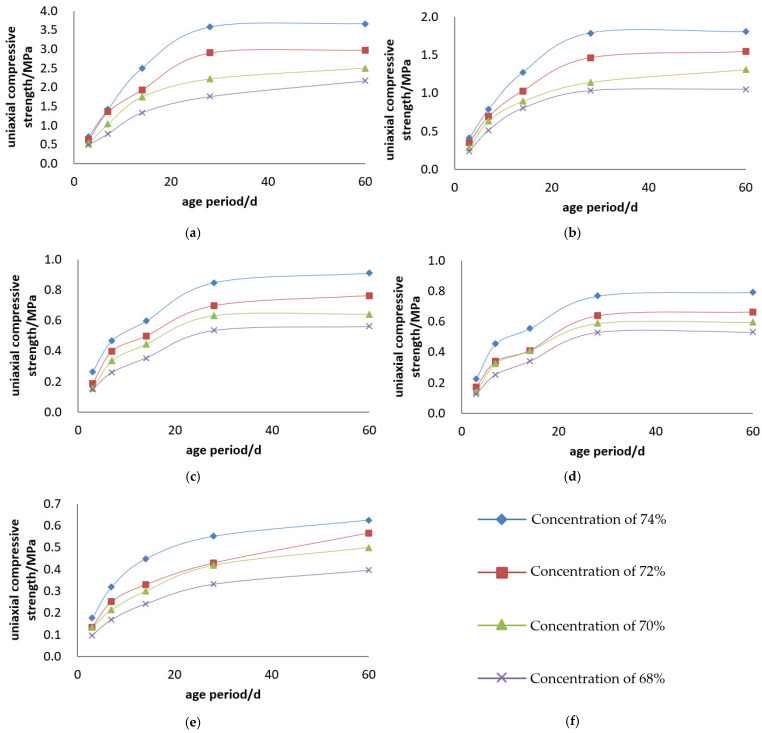
Uniaxial compressive strength curves of Jinyuan 32.5# cement + tailings slurry specimens: (**a**) cement–sand ratio of 1:4, (**b**) cement–sand ratio of 1:6, (**c**) cement–sand ratio of 1:10, (**d**) cement–sand ratio of 1:12, (**e**) cement–sand ratio of 1:15 and (**f**) legends.

**Figure 4:**
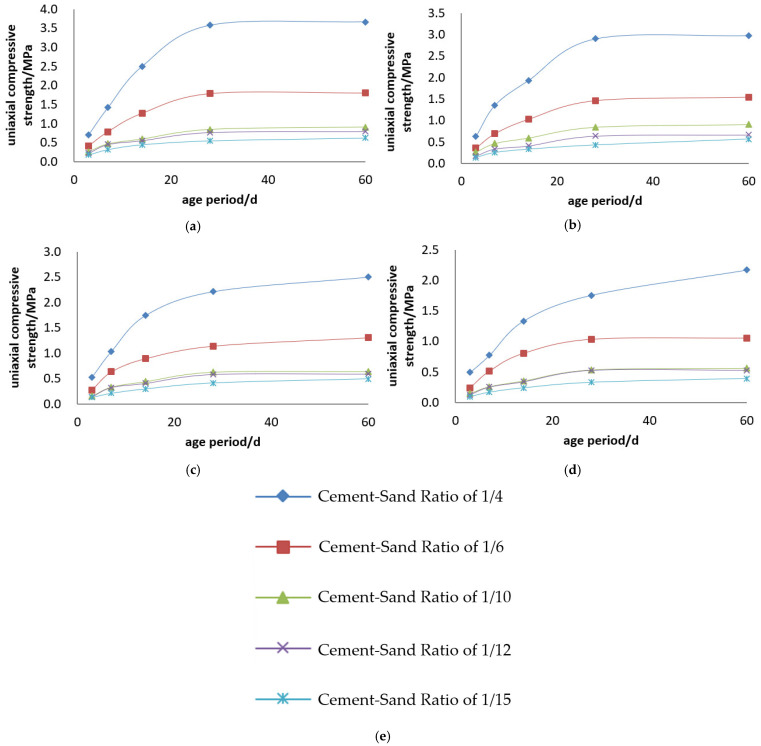
Uniaxial compressive strength curves of Jinyuan 32.5# cement + tailings slurry specimens: (**a**) concentration of 74%, (**b**) concentration of 72%, (**c**) concentration of 70%, (**d**) concentration of 68% and (**e**) legends.

The uniaxial compressive strength of the Jinyuan 32.5# Cement + tailings slurry specimens is presented, and the compressive strength curves are illustrated in Figure 3 and Figure 4.

Backfill slurry concentration is another decisive factor affecting the strength of backfill specimens. At a constant cement–sand ratio, the uniaxial ultimate compressive strength of the specimens increases with the rise in backfill slurry concentration. For example, for backfill with a cement–sand ratio of 1:4, the 3-day uniaxial compressive strength is 0.496 MPa at a concentration of 68%, and 0.705 MPa at 74%—the strength at 74% concentration is 1.4 times that at 68%, increasing to 2.0 times at 28 days. For backfill with a cement–sand ratio of 1:10, the 3-day uniaxial compressive strength is 0.149 MPa at 68% concentration and 0.266 MPa at 74%—the strength at 74% concentration is 1.8 times that at 68% at 3 days, and 1.6 times at 28 days. Based on the test results, to ensure the backfill slurry does not segregate or stratify after being placed in the stope while maintaining good fluidity, the weight concentration of the backfill slurry should be greater than 68%.

Cement dosage is the decisive factor determining the strength of backfill specimens. At a constant backfill slurry concentration, the uniaxial ultimate compressive strength of the specimens increases with the increase in cement dosage. For example, for slurry with a concentration of 74%, the 3-day uniaxial compressive strength of specimens is 0.705 MPa at a cement–sand ratio of 1:4 and 0.177 MPa at 1:15—the strength of specimens with a 1:4 ratio is 4.0 times that of those with a 1:15 ratio at 3 days, and 6.5 times at 28 days. For slurry with a concentration of 70%, the 3-day uniaxial compressive strength of specimens is 0.531 MPa at a cement–sand ratio of 1:4 and 0.133 MPa at 1:15—the strength of specimens with a 1:4 ratio is 4.0 times that of those with a 1:15 ratio at 3 days, and 5.3 times at 28 days.

#### 3.1.2. Kunlun Mountain 42.5# Cement

**Figure 5:**
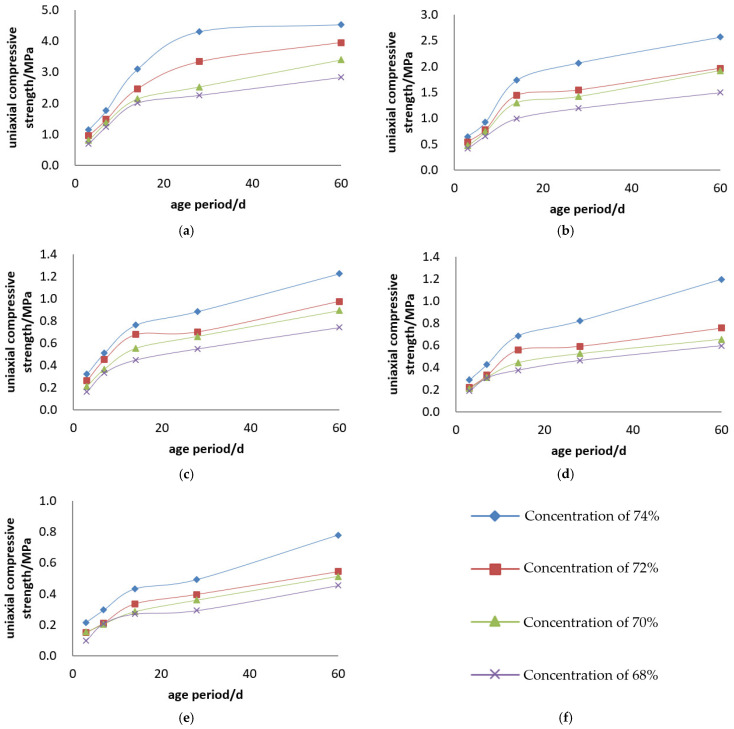
Uniaxial compressive strength curves of Kunlun Mountain 42.5# cement + tailings slurry specimens: (**a**) cement–sand ratio of 1:4, (**b**) cement–sand ratio of 1:6, (**c**) cement–sand ratio of 1:10, (**d**) cement–sand ratio of 1:12, (**e**) cement–sand ratio of 1:15 and (**f**) legends.

**Figure 6:**
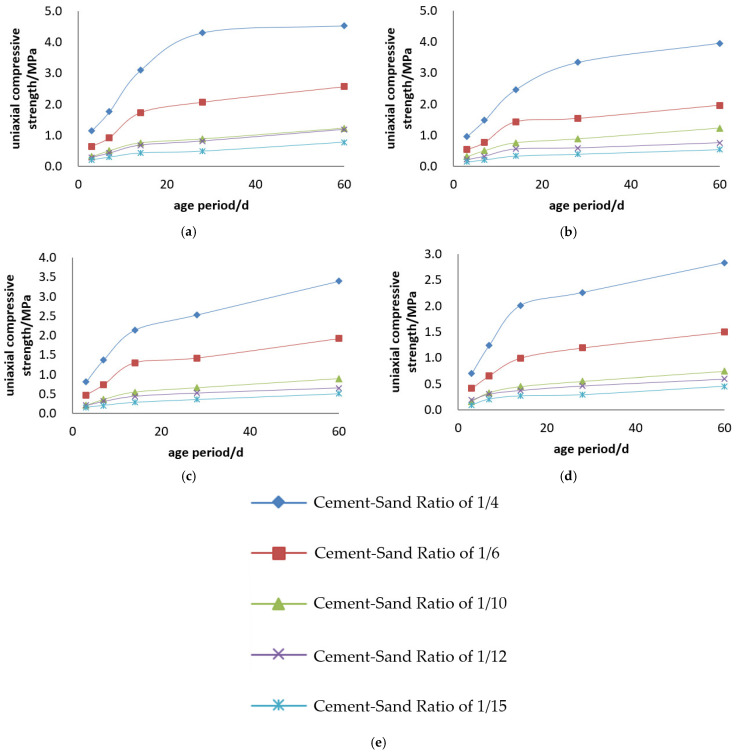
Uniaxial compressive strength curves of Kunlun Mountain 42.5# cement + tailings slurry specimens: (**a**) concentration of 74%, (**b**) concentration of 72%, (**c**) concentration of 70%, (**d**) concentration of 68% and (**e**) legends.

The uniaxial compressive strength and compressive strength curves of the Kunlun Mountain 42.5# cement + tailings slurry specimens are shown in Figure 5 and Figure 6.

Backfill slurry concentration is another decisive factor determining the strength of backfill specimens. When the cement–sand ratio is constant, the uniaxial ultimate compressive strength of the specimens increases with the rise in backfill slurry concentration. For example, regarding the backfill with a cement–sand ratio of 1:4, its 3-day uniaxial compressive strength is 0.697 MPa at a concentration of 68%, while reaching 1.146 MPa at 74%—at 3 days, the strength of specimens with 74% concentration is 1.6 times that of those with 68%, and this ratio rises to 1.9 times at 28 days. For the backfill with a cement–sand ratio of 1:10, its 3-day uniaxial compressive strength is 0.161 MPa at 68% concentration and 0.320 MPa at 74%—at 3 days, the strength of specimens with 74% concentration is 2.2 times that of those with 68%, and this ratio reaches 1.7 times at 28 days.

Cement dosage is the decisive factor for the strength of backfill specimens. When the backfill slurry concentration is constant, the uniaxial ultimate compressive strength of the specimens increases with the increase in cement dosage. For instance, for the slurry with a concentration of 74%, the 3-day uniaxial compressive strength of specimens is 1.146 MPa at a cement–sand ratio of 1:4 and 0.216 MPa at 1:15—at 3 days, the strength of specimens with a 1:4 ratio is 5.3 times that of those with a 1:15 ratio, and this multiple reaches 8.8 times at 28 days. For the slurry with a concentration of 70%, the 3-day uniaxial compressive strength of specimens is 0.807 MPa at a cement–sand ratio of 1:4 and 0.153 MPa at 1:15—at 3 days, the strength of specimens with a 1:4 ratio is 5.3 times that of those with a 1:15 ratio, and this multiple reaches 7 times at 28 days.

#### 3.1.3. Boshushan 32.5# Cement

**Figure 7:**
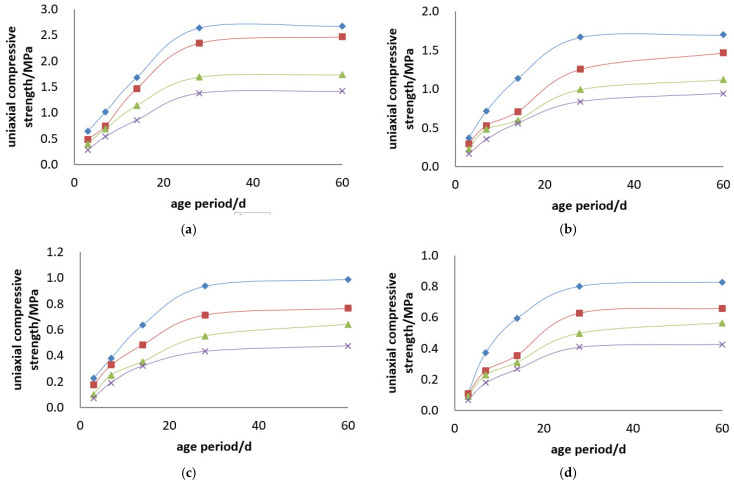

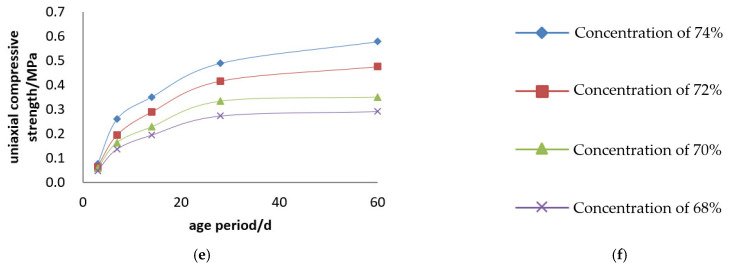
Uniaxial compressive strength curves of Boshushan 32.5# cement + tailings slurry specimens: (**a**) cement–sand ratio of 1:4, (**b**) cement–sand ratio of 1:6, (**c**) cement–sand ratio of 1:10, (**d**) cement–sand ratio of 1:12, (**e**) cement–sand ratio of 1:15 and (**f**) legends.

**Figure 8:**
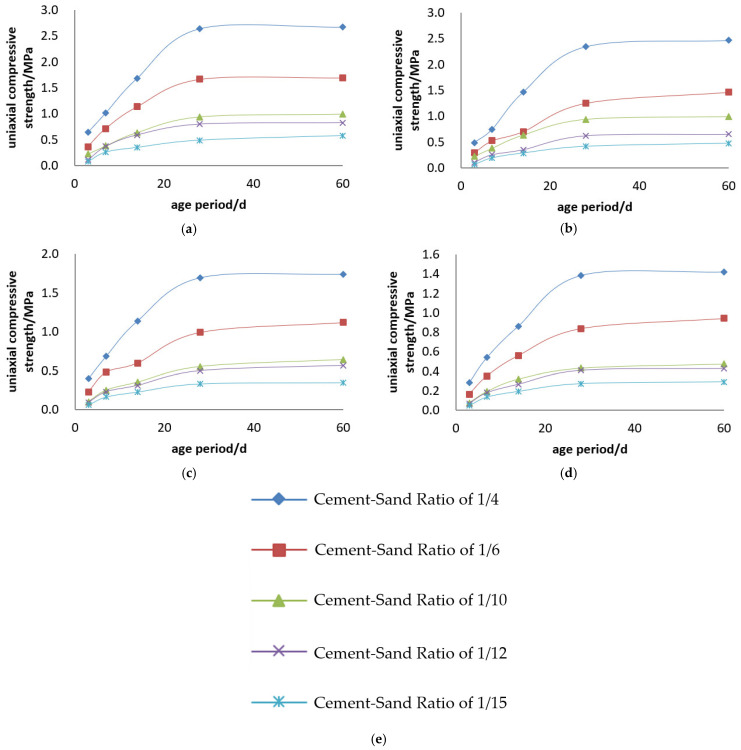
Uniaxial compressive strength curves of Boshushan 32.5# cement + tailings slurry specimens: (**a**) concentration of 74%, (**b**) concentration of 72%, (**c**) concentration of 70%, (**d**) concentration of 68% and (**e**) legends.

The uniaxial compressive strength and uniaxial compressive strength curves of the Boshushan 32.5# cement + tailings slurry specimens are shown in Figure 7 and Figure 8.

Backfill Slurry Concentration: Backfill slurry concentration is another decisive factor determining the strength of backfill specimens. When the cement–sand ratio is constant, the uniaxial ultimate compressive strength of the specimens increases with the rise in backfill slurry concentration. For example, for the backfill with a cement–sand ratio of 1:4, the 3-day uniaxial compressive strength is 0.283 MPa at a concentration of 68%, while it reaches 0.642 MPa at 74%. At 3 days, the strength of the specimens with a concentration of 74% is 2.3 times that of those with a concentration of 68%, and this ratio reaches 1.9 times at 28 days. For the backfill with a cement–sand ratio of 1:10, the 3-day uniaxial compressive strength is 0.073 MPa at a concentration of 68%, and 0.226 MPa at a concentration of 74%. At 3 days, the strength of the specimens with a concentration of 74% is 3.1 times that of those with a concentration of 68%, and 2.2 times at 28 days.

Cement–sand Ratio: Cement dosage is the decisive factor for the strength of backfill specimens. When the backfill slurry concentration is constant, the uniaxial ultimate compressive strength of the specimens increases with the increase in cement dosage. For instance, for the slurry with a concentration of 74%, the 3-day uniaxial compressive strength of the specimens is 0.642 MPa when the cement–sand ratio is 1:4, and 0.077 MPa when the ratio is 1:15. At 3 days, the strength of the specimens with a cement–sand ratio of 1:4 is 8.4 times that of those with a ratio of 1:15, and this multiple reaches 5.4 times at 28 days. For the slurry with a concentration of 70%, the 3-day uniaxial compressive strength of the specimens is 0.397 MPa when the cement–sand ratio is 1:4, and 0.059 MPa when the ratio is 1:15. At 3 days, the strength of the specimens with a cement–sand ratio of 1:4 is 6.8 times that of those with a ratio of 1:15, and 5.1 times at 28 days.

In summary, both slurry concentration and cement-to-sand ratio act as fundamental drivers of CTB strength development. While higher cement dosage provides more hydration products to bind the matrix, increased concentration optimizes the internal structure by reducing porosity and shortening inter-particle distances. The observed strength growth—particularly the more pronounced impact of cement content at early ages—underscores the necessity of balancing these two factors to achieve both rapid cycle times and long-term stability in mine backfilling operations.

#### 3.1.4. Boshushan 42.5# Cement

**Figure 9:**
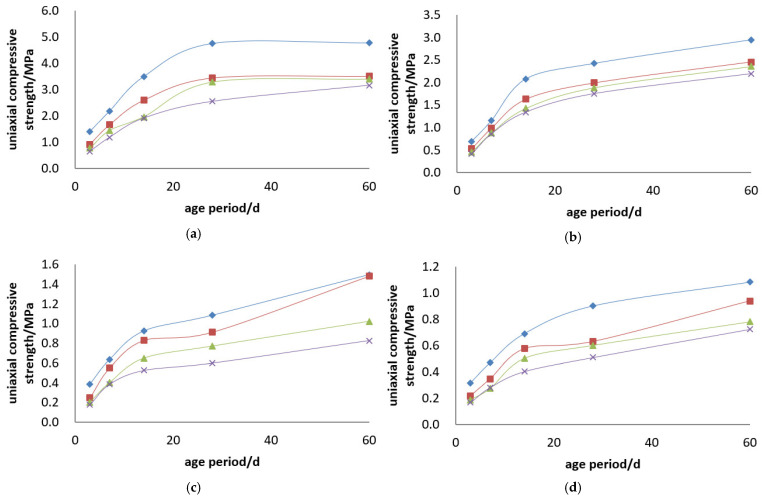

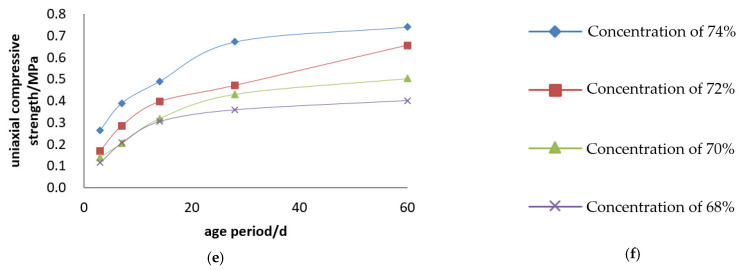
Uniaxial compressive strength curves of Boshushan 42.5# cement + tailings slurry specimens: (**a**) cement–sand ratio of 1:4, (**b**) cement–sand ratio of 1:6, (**c**) cement–sand ratio of 1:10, (**d**) cement–sand ratio of 1:12, (**e**) cement–sand ratio of 1:15 and (**f**) legends.

**Figure 10:**
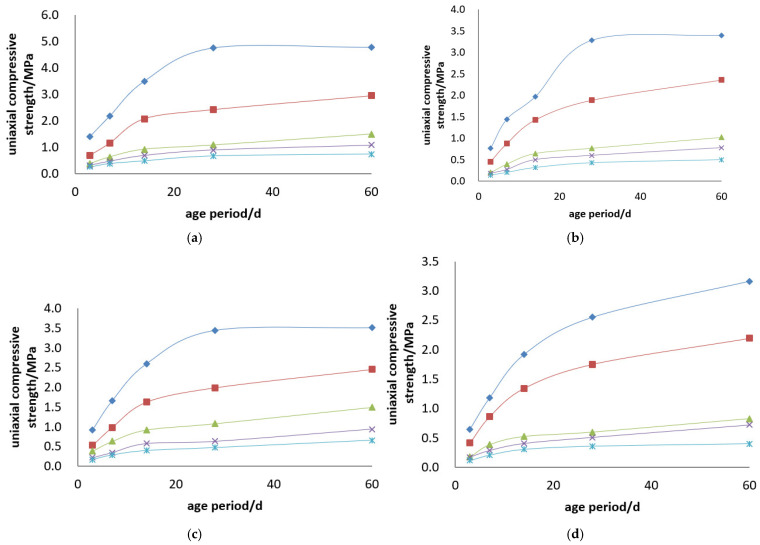

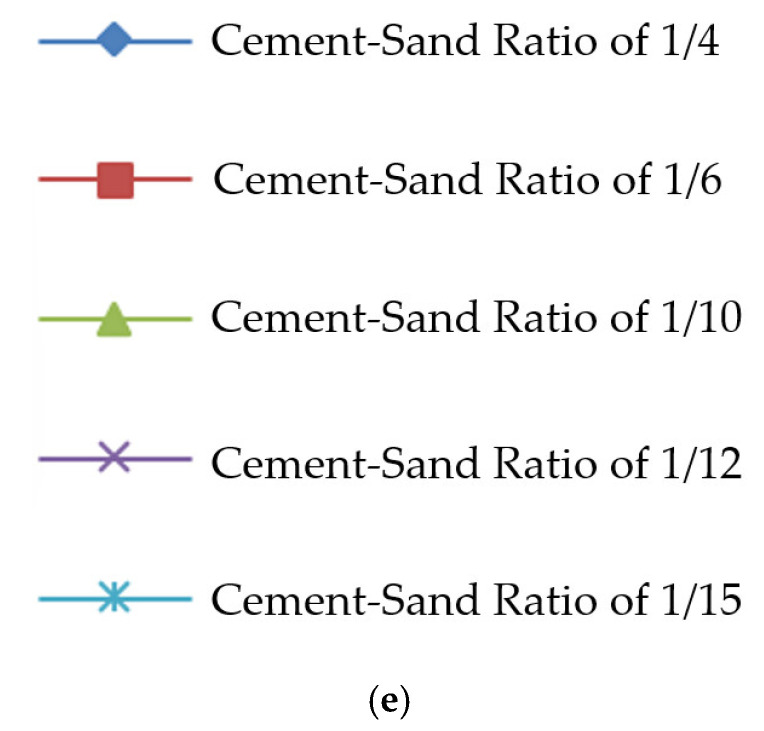
Uniaxial compressive strength curves of Boshushan 42.5# cement + tailings slurry specimens: (**a**) concentration of 74%, (**b**) concentration of 72%, (**c**) concentration of 70%, (**d**) concentration of 68% and (**e**) legends.

The uniaxial compressive strength and uniaxial compressive strength curves of the Boshushan 42.5# cement + tailings slurry specimens are shown in Figure 9 and Figure 10.

Backfill Slurry Concentration: Backfill slurry concentration is another decisive factor determining the strength of backfill specimens. When the cement–sand ratio is constant, the uniaxial ultimate compressive strength of the specimens increases with the rise in backfill slurry concentration. For example, for the backfill with a cement–sand ratio of 1:4, the 3-day uniaxial compressive strength is 0.649 MPa at a concentration of 68%, while it reaches 1.407 MPa at 74%. The strength of the specimens with a concentration of 74% is 2.2 times that of those with a concentration of 68%, and this ratio reaches 1.9 times at 28 days. For the backfill with a cement–sand ratio of 1:10, the 3-day uniaxial compressive strength is 0.176 MPa at a concentration of 68%, and 0.387 MPa at a concentration of 74%. At 3 days, the strength of the specimens with a concentration of 74% is 2.2 times that of those with a concentration of 68%, and 1.8 times at 28 days.

Cement-to-Sand Ratio: Cement dosage is the primary determinant of backfill strength. At a constant slurry concentration, uniaxial compressive strength (UCS) increases significantly with higher cement content. For a 74% concentration, the 3-day UCS at a 1:4 ratio (1.407 MPa) is 5.3 times higher than at 1:15 (0.264 MPa), with this disparity reaching 7.1 times by 28 days. Similarly, at 70% concentration, the 3-day UCS for a 1:4 ratio (0.770 MPa) is 5.5 times that of a 1:15 ratio (0.141 MPa), expanding to 7.6 times at 28 days.

### 3.2. Slump Determination Test of Backfill Slurry

#### 3.2.1. Slump Test Results of Unclassified Tailings Slurry

**Figure 11:**
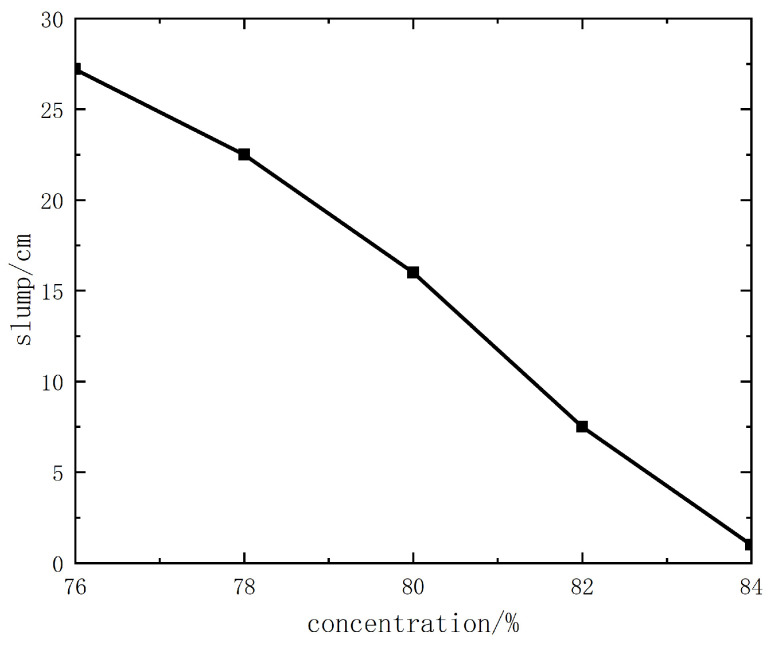
Unclassified tailings slump curve diagram.

The test results of the unclassified tailings slurry are shown in detail in Figure 11. From the slump curve, when the slurry concentration is lower than 80%, the slump value increases rapidly; when the slurry concentration is 82% or higher, the slump is ≤7.5 cm, and the backfill slurry is dry-hard, making it impossible for pipeline transportation. When the concentration of the unclassified tailings slurry without cement is 78%, the slump is 22.5 cm, and pipeline transportation can only be carried out with pressure from a piston pump. When the concentration is 76% or lower, the slump is above 27.2 cm, enabling gravity flow pipeline transportation. When the slurry concentration is above 70%, the slurry has good workability and water retention performance, with a small amount of bleeding and no easy segregation, which is more conducive to transportation. 

#### 3.2.2. Slump Test Results of Jinyuan 32.5# Cement + Unclassified Tailings Slurry

**Figure 12:**
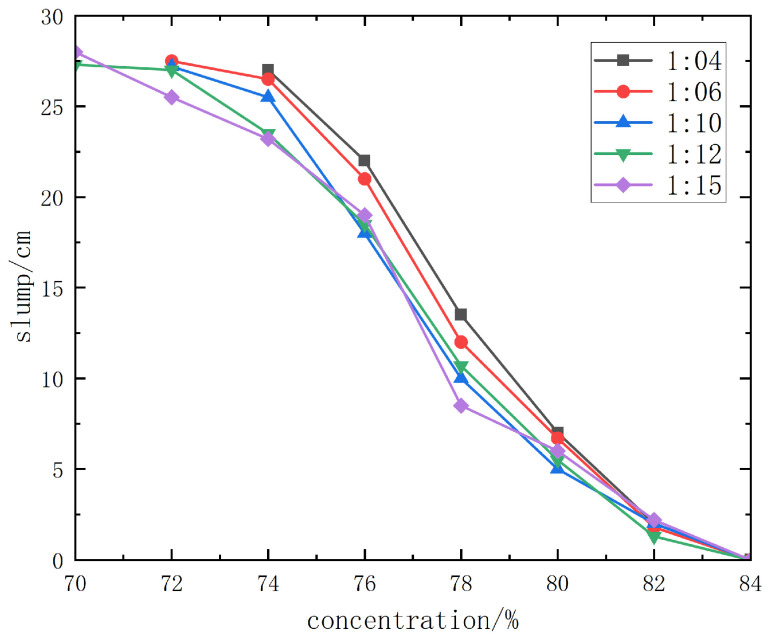
Slump test results curve diagram of Jinyuan 32.5# cement.

The slump test results of Jinyuan 32.5# cement + unclassified tailings slurry are shown in detail in Figure 12.

From the slump curve diagram (Figure 11), when the slurry concentration is 78% or higher, the slump of slurries with different cement–sand ratios is ≤8.5~13.5 cm, and the backfill slurry is dry-hard, making pipeline transportation impossible. When the slurry concentration is 76%, the slump is 18~22 cm, and pipeline transportation can only be carried out with pressure from a piston pump. When the concentration is 74% or lower, the slump is above 23.2 cm, the slurry has good fluidity, and gravity flow pipeline transportation can be realized.

From the slump and spreading morphology of unclassified tailings slurries with various mix ratios and concentrations, it can be observed that when the slurry concentration is higher than 68%, the slurry has good water retention performance, without adverse phenomena such as segregation of coarse and fine particles, dehydration, or excessive bleeding. Therefore, the backfill slurry concentration is initially set to 70%~74%, with a corresponding slump of 23.2 cm completely spreading.

#### 3.2.3. Slump Test Results of Kunlun 42.5# Cement + Unclassified Tailings Slurry

**Figure 13:**
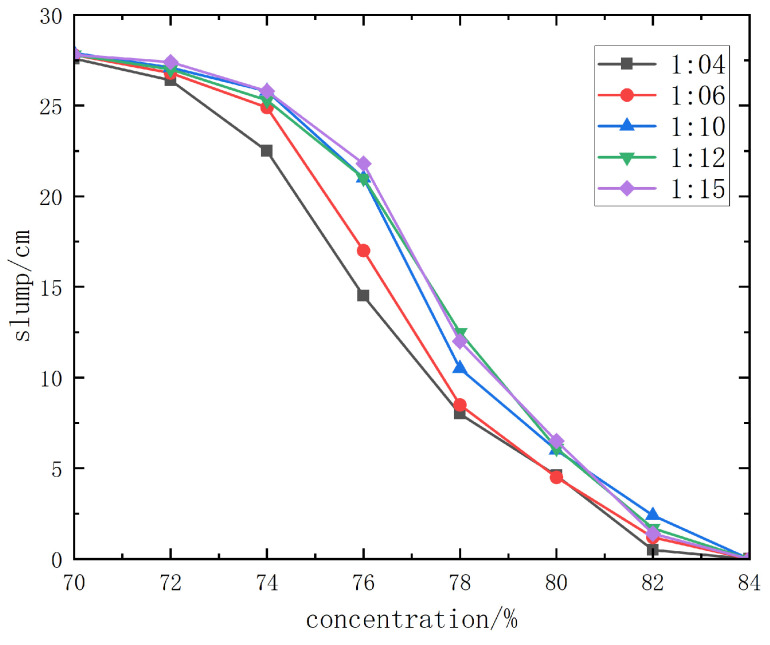
Slump test results curve diagram of Kunlun 42.5# cement.

The slump test results of Kunlun 42.5# cement + unclassified tailings slurry are shown in detail in Figure 13.

From the slump curve diagram, when the slurry concentration is 78% or higher, the slump of slurries with different cement–sand ratios is ≤8~12.5 cm, and the backfill slurry is dry-hard, making pipeline transportation impossible. When the slurry concentration is 76%, the slump of slurries with different cement–sand ratios is 14.5~21.8 cm, and pipeline transportation can only be carried out with pressure from a piston pump. When the concentration is 74% or lower, the slump is above 22.5 cm, the slurry has good fluidity, and gravity flow pipeline transportation can be realized.

The fluidity improves significantly with the decrease in concentration, but this will inevitably lead to the reduction in backfill strength. From the slump and spreading morphology of unclassified tailings slurries with various mix ratios and concentrations, it can be observed that when the slurry concentration is higher than 68%, the slurry has good water retention performance, without adverse phenomena such as segregation of coarse and fine particles, dehydration, or excessive bleeding. Therefore, the backfill slurry concentration is initially set to 68%~74%, with a corresponding slump of 22.5 cm completely spreading.

#### 3.2.4. Slump Test Results of Boshushan 32.5# Cement + Unclassified Tailings Slurry

**Figure 14:**
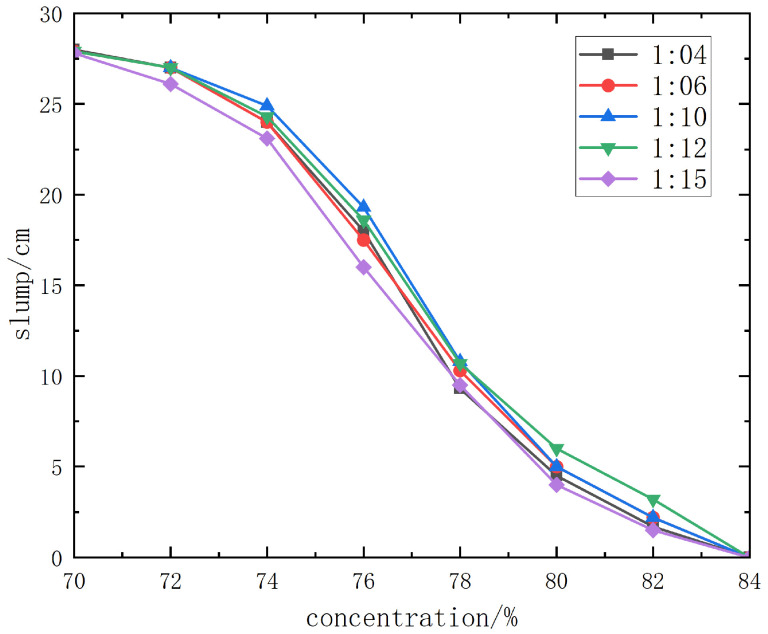
Slump test results curve diagram of Boshushan 32.5# cement.

The slump test results of Boshushan 32.5# cement + unclassified tailings slurry are shown in detail in Figure 14.

From the slump curve diagram, when the slurry concentration is 78% or higher, the slump is ≤9.3~10.8 cm, and the backfill slurry is dry-hard, making pipeline transportation impossible. When the concentration of the unclassified tailings slurry without cement is 76%, the slump is 16~19.3 cm, and pipeline transportation can only be carried out with pressure from a piston pump. When the concentration is 74% or lower, the slump is above 23.1 cm, the slurry has good fluidity, and gravity flow pipeline transportation can be realized.

The fluidity improves significantly with the decrease in concentration, but this will inevitably lead to the reduction in backfill strength. From the slump and spreading morphology of unclassified tailings slurries with various mix ratios and concentrations, it can be observed that when the slurry concentration is higher than 70%, the slurry has good water retention performance, without adverse phenomena such as segregation of coarse and fine particles, dehydration, or excessive bleeding. Therefore, the backfill slurry concentration is initially set to 70%~74%, with a corresponding slump of 23.1 cm completely spreading.

#### 3.2.5. Slump Test Results of Boshushan 42.5# Cement + Unclassified Tailings Slurry

**Figure 15:**
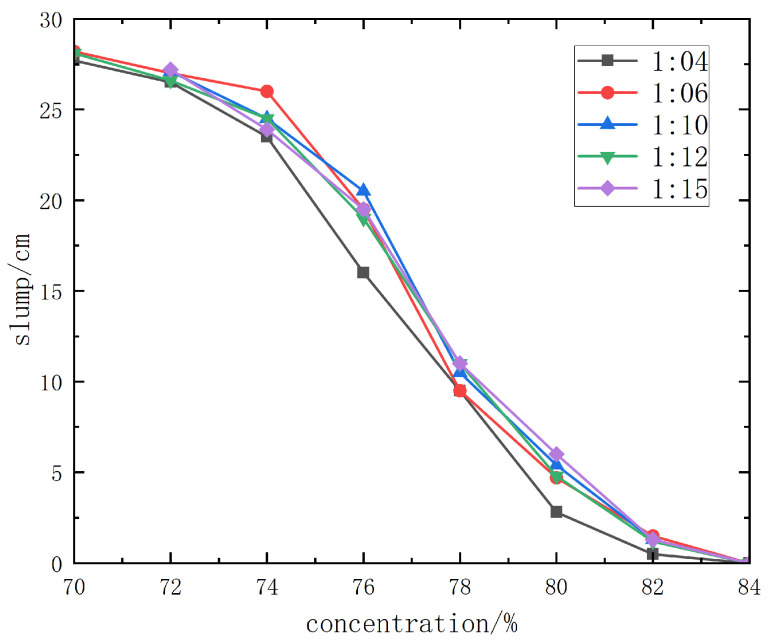
Slump test results curve diagram of Boshushan 42.5# cement.

The slump test results of Boshushan 42.5# cement + unclassified tailings slurry are shown in detail in Figure 15.

From the slump curve diagram, when the slurry concentration is 78% or higher, the slump of slurries with different cement–sand ratios is ≤9.5~11.0 cm, and the backfill slurry is dry-hard, making pipeline transportation impossible. When the slurry concentration is 76%, the slump of slurries with different cement–sand ratios is 16~20.5 cm, and pipeline transportation can only be carried out with pressure from a piston pump. When the concentration is 74% or lower, the slump is above 23.5 cm, the slurry has good fluidity, and gravity flow pipeline transportation can be realized.

From the slump and spreading morphology of unclassified tailings slurries with various mix ratios and concentrations, it can be observed that when the slurry concentration is higher than 70%, the slurry has good water retention performance, without adverse phenomena such as segregation of coarse and fine particles, dehydration, or excessive bleeding. Therefore, the backfill slurry concentration is initially set to 70%~74%, with a corresponding slump of 23.5 cm completely spreading.

## 4. Discussion

### 4.1. In-Depth Analysis of Slump and Workability of Backfill Slurry

This study systematically evaluated the influence of different cement types, grades, and slurry parameters on the workability of backfill slurry through slump tests. The results indicate that slurry concentration is the primary factor controlling its fluidity, which is closely related to the interaction mechanisms of solid particles in a suspension. When the concentration is below 74%, the slurry exhibits good fluidity (slump ≥ 22.5 cm), enabling gravity-flow transportation. However, when the concentration increases to 76% or above, the slurry rapidly transitions to a dry and stiff state, with a sharp decline in fluidity. This phenomenon can be explained from a rheological perspective: at high concentrations, the distance between tailings particles decreases, inter-particle friction and collision intensify, and the water film thickness becomes insufficient, leading to a significant increase in the slurry’s yield stress and plastic viscosity, thereby resulting in loss of fluidity.

Although the effects of cement brand, grade, and cement-to-tailings ratio on slump are relatively limited, they play crucial roles in the formation and stability of the slurry’s internal structure. This study found that all slurry mixes with high concentrations (≥68~70%) demonstrated excellent water retention and stability, with no bleeding or segregation. This suggests that within the optimized concentration range, cement hydration products and tailings particles form a stable spatial network structure that effectively encapsulates and locks in free water. This characteristic is crucial for long-distance pipeline transportation, preventing pipe blockage and ensuring the homogeneity of the backfill.

### 4.2. Strength Development Patterns and Microscopic Mechanism Analysis

The strength test results clearly reveal that the cement-to-tailings ratio is the most dominant factor affecting backfill strength, with its influence significantly stronger than that of slurry concentration. Increasing cement dosage can substantially enhance strength, particularly the development of early strength. This is primarily attributed to the cementing effect of the cement hydration reaction: hydration products (such as C-S-H gel, ettringite) form, grow, and interweave between and on the surfaces of tailings particles, creating a load-bearing skeletal structure. A higher cement-to-tailings ratio means more cementitious material per unit volume, leading to the formation of a denser cementitious network and consequently higher macroscopic strength.

The influence of cement grade is also significant. Under comparable conditions, 42.5# cement generally demonstrates superior strength performance compared to 32.5# cement, along with a lower cement cost per unit strength, highlighting its comprehensive advantages. This is mainly because 42.5# cement typically has finer particle size, higher content of active minerals (e.g., C3S), and a more optimized particle size distribution, enabling a faster and more complete hydration reaction and generating a greater quantity and stronger quality of cementitious products.

The mechanism by which slurry concentration affects strength is related to the compactness and internal porosity of the hardened slurry. With a fixed cement-to-tailings ratio, a higher concentration implies a greater content of solid particles per unit volume, resulting in lower porosity and a denser structure in the final hardened body. The data from this study show that increasing the concentration from 68% to 74% can enhance strength by 1.4 to 3.1 times (depending on the cement-to-tailings ratio). This reveals a pattern, namely that higher solid content contributes to the formation of a denser microstructure.

### 4.3. Scientific Basis for Optimized Mix Proportion and Engineering Implications

Integrating the slump and strength results, this study recommends using 42.5# cement, controlling the slurry concentration within the range of 70%~74%, and determining an economically rational cement-to-tailings ratio based on the required underground strength grade. This optimized range scientifically balances the inherent contradiction between “transportability” and “strength performance.” Concentrations below 70%, while offering better fluidity, lead to a significant reduction in strength and may increase the risk of bleeding. Concentrations above 76% make transportation difficult or even impossible. From a microscopic mechanism perspective, the concentration range of 70%~74% achieves the maximization of solid content while ensuring a sufficient lubricating water film for flow, laying the foundation for forming a high-strength, low-porosity hardened body.

It is noteworthy that this study is primarily based on macroscopic mechanical testing. To gain deeper insights into the mechanisms underlying the performance differences of backfill with different mix proportions, future research could employ advanced instrumental analysis techniques at the micro-scale. For example,

(1) Scanning Electron Microscopy (SEM): Could directly observe the morphology and distribution of hydration products and their interfacial bonding with tailings particles under different cement-to-tailings ratios, concentrations, and cement types, revealing the microstructural roots of strength differences.
(2) X-ray Diffraction (XRD): Could quantitatively analyze the phase composition and content of hydration products in backfill with different mixes, particularly the proportion of cementitious C-S-H gel versus potentially harmful products (e.g., delayed ettringite), assessing long-term stability.
(3) Mercury Intrusion Porosimetry (MIP) or Nitrogen Adsorption: Could accurately measure porosity, pore size distribution, and tortuosity, establishing quantitative relationships between microscopic pore structure and macroscopic properties like strength and permeability.

Through the aforementioned microanalyses, clear correlations can be established between macroscopic properties (strength, fluidity) and microscopic characteristics (structure, phase, pores), moving beyond merely knowing “what” happens to understanding “why” it happens. This provides a more solid scientific basis for the precise design and performance control of backfill materials. The optimal mix proportion range established in this study represents the optimal solution derived from the synergistic effect of macroscopic performance, microscopic formability, and economic efficiency, offering clear guiding value for full tailings backfill practices in similar mines.

## 5. Conclusions

This study systematically analyzed the physicochemical properties, fluidity, and mechanical strength of full tailings backfill materials in a lead–zinc mine, revealing the evolution patterns of backfill performance under the coupling effects of multiple factors. Experimental analysis demonstrates that the strength of full tailings backfill is not controlled by a single factor, but is the result of the synergistic effects of cement type, cement-to-tailings ratio, slurry concentration, and curing age. It is found that the cement-to-tailings ratio is the decisive factor for the final strength, while the slurry concentration is critical to early-age strength development; specifically, increasing the concentration from 68% to 74% can yield a significant strength improvement of 1.4 to 3.1 times. Furthermore, this work defined the critical concentration range of 68–74% to meet the requirements of pipeline transportation, ensuring favorable fluidity while preventing segregation, thereby balancing the trade-off between pumpability and mechanical performance.

Regarding research innovation, unlike conventional tests focused on a single cement type, this study incorporated practical logistics costs to compare the suitability of various cement brands and strength grades in a full tailings environment. The results suggest that 42.5# cement offers superior techno-economic cost-effectiveness, as it provides a broader strength coverage and excellent early-age strength at lower dosages. This finding not only optimizes the backfilling process parameters for the mine but also provides a scientifically based proportioning scheme for full tailings utilization.

Although reliable process parameters were obtained through large-scale macroscopic mechanical tests, due to experimental limitations, the microscopic gelation mechanism and long-term environmental stability of full tailings require further investigation using microscopic techniques such as SEM and XRD. Nevertheless, this work validates the significant potential of Cemented Tailings Backfill (CTB) technology in achieving zero waste discharge and constructing “green mines.” The research outcomes not only directly guide the efficient and safe backfilling practices of the lead–zinc mine but also provide a crucial theoretical basis and technical reference for the resource utilization of full tailings in metal mines with similar physical properties.

## Figures and Tables

**Table 1:** Test results of physical performance indicators of unclassified tailings.

Test Item	Specific Gravity	Loose Dry Bulk Density/(t/m^3^)	Dense Dry Bulk Density/(t/m^3^)	Loose Porosity/%	Dense Porosity/%
Data	2.73	1.367	1.908	51.52	32.34

**Table 2:** Factors and levels of cement-unclassified tailings slurry specimen tests.

Serial Number	Cement Type	Concentration (%)	Cement–Sand Ratio	Curing Age (Days)
1	Jinyuan 32.5# Cement	68	1:4	3
2	Kunlun Mountain 42.5# Cement	70	1:6	7
3	Baishu Mountain 32.5# Cement	72	1:10	14
4	Baishu Mountain 42.5# Cement	74	1:12	28
5			1:15	60

## Data Availability

The original contributions presented in the study are included in the article; further inquiries can be directed to the corresponding author.
